# Antiviral Activity of an Endogenous Parvoviral Element

**DOI:** 10.3390/v15071420

**Published:** 2023-06-23

**Authors:** Angelica Bravo, Leandro Fernández-García, Rodrigo Ibarra-Karmy, Gonzalo A. Mardones, Luis Mercado, Fernando J. Bustos, Robert J. Gifford, Gloria Arriagada

**Affiliations:** 1 Instituto de Ciencias Biomedicas, Facultad de Medicina y Facultad de Ciencias de la Vida, Universidad Andres Bello, Santiago 8370071, Chile; ange.bravo@uandresbello.edu (A.B.); leandrofg1990@gmail.com (L.F.-G.); r.ibarrakarmy@gmail.com (R.I.-K.); fernando.bustos@unab.cl (F.J.B.); 2 Facultad de Medicina y Ciencia, Universidad San Sebastián, Valdivia 5110466, Chile; gonzalo.mardonesc@uss.cl; 3 Instituto de Biología, Facultad de Ciencias, Pontificia Universidad Católica de Valparaíso, Valparaíso 2373223, Chile; luis.mercado@pucv.cl; 4 Centre for Virus Research, MRC-University of Glasgow, 464 Bearsden Rd, Bearsden, Glasgow G61 1QH, UK

**Keywords:** parvovirus, immunity, evolution, paleovirology, endogenous viral elements

## Abstract

Endogenous viral elements (EVEs) are genomic DNA sequences derived from viruses. Some EVEs have open reading frames (ORFs) that can express proteins with physiological roles in their host. Furthermore, some EVEs exhibit a protective role against exogenous viral infection in their host. Endogenous parvoviral elements (EPVs) are highly represented in mammalian genomes, and although some of them contain ORFs, their function is unknown. We have shown that the locus *EPV-Dependo.43-ODegus*, an EPV with an intact ORF, is transcribed in *Octodon degus* (degu). Here we examine the antiviral activity of the protein encoded in this EPV, named DeRep. DeRep was produced in bacteria and used to generate antibodies that recognize DeRep in western blots of degu tissue. To test if DeRep could protect against exogenous parvovirus, we challenged cells with the minute virus of mice (MVM), a model autonomous parvovirus. We observed that MVM protein expression, DNA damage induced by replication, viral DNA, and cytopathic effects are reduced when DeRep is expressed in cells. The results of this study demonstrate that DeRep is expressed in degu and can inhibit parvovirus replication. This is the first time that an EPV has been shown to have antiviral activity against an exogenous virus.

## 1. Introduction

The genomes of extant species contain numerous DNA sequences derived from viruses. It is proposed that these endogenous viral elements (EVEs) arise when infection of germline cells (i.e., gametes or early embryonic cells) leads to the integration of viral sequences into the chromosomal DNA of ancestral organisms, such that they are subsequently inherited from parent to offspring as novel genes [1]. Most EVEs are derived from viruses that circulated millions of years ago and therefore represent the viral fossil record, providing unique insight into the long-term evolutionary interactions between viruses and cells [2,3,4]. Because retroviruses integrate into genomic DNA as an obligate step in their replication, most EVE sequences found in mammalian genomes are derived from retroviruses (family *Retroviridae*), compared to a relatively small number of EVE sequences derived from non-retroviral virus families [1,5,6,7], whose integration is thought to occur only anomalously through non-homologous recombination (DNA viruses) or retrotransposition of viral mRNA (DNA and RNA viruses) [8,9,10].

Genomic and experimental research has revealed that some of these ‘horizontally transferred’ viral sequences have been co-opted or exapted to perform physiologically relevant functions. In mammals, EVEs have been shown to be relevant for cell function, embryonic development, and antiviral immunity [11,12,13,14,15,16,17]. While most examples involve endogenous retroviruses (ERVs), potentially co-opted/exapted EVEs derived from non-retroviral viruses have also been identified and investigated [16,17]. For example, endogenous bornavirus-like nucleoprotein (EBLN) has been shown to be coopted and expressed as both RNA and proteins in several species. In humans, hsEBLN-1 is expressed in the testis and brain [18]. Although this gene contains an intact ORF, it is proposed to function in gene regulation as a non-coding RNA (ncRNA) [16,18], while hsEBLN-2 encodes the mitochondrial E2 protein that has been shown to interact with apoptosis-related host proteins, affecting cell viability [17]. In the thirteen-lined ground squirrel (*Ictidomys tridecemlineatus*), an EBLN encodes an intact nucleoprotein that co-localizes in the nucleus with viral factories and inhibits in vitro replication of the Borna disease virus (BDV) [15].

Parvoviruses (family *Parvoviridae*) are well represented among the non-retroviral EVEs documented in mammals [1,4,7,19,20,21,22,23,24]. Parvoviruses are small, non-enveloped viruses of icosahedral symmetry. They have a linear, single-stranded DNA (ssDNA) genome of 4 to 6.3 kilobases (kb) in length [25] and encode at least two major open reading frames (ORFs) that are expressed to produce non-structural (NS or Rep) proteins and structural (VP or Capsid) proteins. Parvovirus replication occurs in the nucleus, and occasionally viral genome integration into host chromosomes can happen through non-homologous recombination, possibly facilitated by single-stranded breaks created by the nickase function of Rep [26]. Thus, incorporation of parvovirus DNA into the germline DNA and the formation of endogenous parvoviruses (EPVs) might be expected to occur at a certain frequency as a natural consequence of the biology of these viruses. However, as is the case for any new allele, the majority of EPVs generated in such events will be rapidly eliminated from the gene pool by genetic drift unless they are selected for some reason. Thus, the presence of several independently acquired, fixed EPV insertions in animal genomes is unexpected and suggests that selective pressures may have favored the retention of EPV genes in animal genomes during their evolution. Intriguingly, several EPV loci containing open reading frames (ORFs) capable of expressing complete or almost complete proteins expressed at least as transcripts have been reported [21,22,27].

We have previously shown that an intact EPV locus (*EPV-Dependo.43-ODegus*), derived from the Rep gene of a dependoparvovirus, is transcribed in the degu (*Octodon degus*) [22]. Degus are small rodents endemic to the Chilean Matorral ecoregion, where they live in colonial burrows. They are intelligent social animals that are responsive to human interaction and are often kept as pets. In the present study, we investigate the protein expression and antiviral activity of the protein encoded in this EPV, named DeRep, which is present in degu.

## 2. Materials and Methods

### 2.1. Cell Culture

Human embryonic kidney cells (HEK293T) were used for viral particle production, and mouse fibroblast cells (NIH3T3) were used for infection assays. Both cell lines were maintained at 37 °C with 5% CO_2_ in Dulbecco’s modified Eagle medium (DMEM) supplemented with 10% fetal bovine serum, 100 IU/mL penicillin, and 100 μg/mL streptomycin.

### 2.2. Cloning and Plasmids

The infectious parvovirus molecular clones pdBMVp, kindly provided by Peter Tattersall [28], and pcDNA3xFLAG-DeRep [21] have been described previously. To generate pEGFP-DeRep, the DeRep coding sequence was obtained by digestion of pcDNA3xFLAG-DeRep with *BamH*I and *Xho*I and ligated into pEGFP C1 digested with *Bgl*II and *Sal*I. To obtain the lentiviral plasmid pLVX-3xFLAG-DeRep-Puro, the coding sequence of DeRep, including the FLAG epitope was PCR amplified from pcDNA3xFLAG-DeRep using the primers *Xho*I-FLAGstart-F 5′-tatactcgagatggactacaaagaccatga-3′ and DeRep-*Not*I-R 5′-atatgcggccgcctagagggcgactttttcc-3′, the PCR product was gel purified, digested with *Xho*I and *Not*I and ligated into pLVX-IRES-PURO digested with the same restriction enzymes. The plasmid pcDNA3xFLAG-enRepM9L was previously described [21]. To generate pcDNA3xFLAG-OcenRep, rabbit genomic DNA was amplified using the primers OcRep*BamH*I-F 5′-atggatccatggaagagtatataagggcggct-3′ and OcRep*Xho*I-R 5′-aatctcgagttatcctccgccaagtcttcc-3′. The PCR product was gel purified, digested with *BamH*I and *Xho*I, and ligated into pcDNA3xFLAG digested with the same restriction enzymes. pcDNA3xFLAG-RhTRIM5α was previously described [29].

### 2.3. Generation of Stable Cell Lines

Lentiviruses for transduction were produced by co-transfection of HEK293T cells with 5 µg of pMD.G (encoding the Vesicular Stomatitis Virus Envelope Glycoprotein), 5 µg of p8.91 (encoding for Gal-Pol of HIV-1), 2.5 µg of pRSVRev (encoding rev from HIV-1), and 10 µg of pLVX-IRES-PURO or pLVX-3xFLAG-DeRep-Puro using polyethyleneimine (PEI) 8 mg/mL in a 3:1 proportion. Viruses were harvested 48 h after transfection, filtered (0.45 µm), and used to transduce NIH3T3 cells in the presence of 8 µg/mL Polybrene. Cells were selected for 1 µg/mL of puromycin.

### 2.4. Antibody Generation

pcDNA3xFLAG-DeRep was digested with *BamH*I and *Xho*I. The coding sequence of DeRep was purified and ligated into pGEX4T1, digested with the same restriction enzymes. GST-DeRep was purified from bacteria, and DeRep was obtained by digestion with thrombin. DeRep was used to immunize 2 mice, and serum 120 and 121 were obtained. Serums were then affinity purified using GST-DeRep as bait.

### 2.5. Minute Virus of Mice Production

HEK293T seeded in 150-mm plates were transfected with 10 µg of pdBMVp using PEI 3:1 in fresh media. After 24 h, the media was changed to DMEM with 3% FBS, and cells were cultured until cytopathic effects appeared (days 2–5 post-transfection). Media was collected and filtered to 0.2 µm, and the virus was aliquoted and stored at −80 °C until use.

### 2.6. Viral DNA Quantification

HEK293T cells (6 × 10^4^ cells/well) were seeded in 6-well plates. Twenty-four hours after plating, cells were co-transfected with 1 µg of pcDNA3xFLAG-DeRep, 1 µg of pcDNA3xFLAG-RhTRIM5α, or with empty vector and 200 ng of pdBMVp using Lipofectamine 2000. Forty-eight hours after transfection, media were recovered and filtered, and the monolayer was recovered in PBS for DNA and protein extraction. DNA was purified by gel and PCR clean-up columns (Machery-Nagel). Recovered DNA was used for qPCR assay with Brilliant II SYBER Green kit and primers directed to MVM NS (NS-F 5′-ACCAGCCAGCACAGGCAAATCTATTAT-3′; NS-R 5′-CATTCTGTCTCTGATTGGTTGAGT-3′) and host 18S (18S-F 5′-GTGGAGCGATTTGTCTGGTT-3′; 18S-R 5′-CGCTGAGCCAGTCAGTGTAG-3′). Data are expressed as the MVM DNA amount relative to 18S calculated by the ∆∆Ct method [30]. Protein over-expression was determined by western blotting using anti-FLAG and anti-tubulin, as described below.

### 2.7. Western Blot Assays

To analyze the expression of DeRep in fusion with GFP or FLAG, NIH3T3 cells were transfected with 1 µg of either pEGFP, pEGFP-DeRep, pcDNA3xFLAG, pcDNA3xFLAG-DeRep, pcDNA3xFLAG-enRepM9L, or pcDNA3xFLAG-OcenRep using PEI 3:1. Forty-eight hours later, the cells were lysed in Reporter lysis buffer (Promega, Madison, WI, USA). Samples were then boiled in 5× sodium dodecyl sulfate (SDS) loading buffer, and the proteins were resolved by 10% acrylamide SDS-PAGE. After transfer to PVDF membranes, the blots were probed with mouse anti-Flag (Clone M2, Sigma, Kawasaki, Tokyo), mouse anti-GFP B-2 (Santa Cruz Biotechnology, Santa Cruz, CA, USA), mouse anti-DeRep 120, mouse anti-DeRep 121, or mouse anti-α tubulin (Clone DM1A, Sigma). Secondary antibodies conjugated to HRP and ECL reagents were used for development.

To analyze the expression of DeRep in degu, tissues were obtained from a fresh male (3) or female (3) headless *O. degus* cadaver (kindly donated by Dr. Adrian Palacios from the Universidad de Valparaiso, Chile). All experiments were performed according to the protocol approved by the Bioethical Committee of Universidad Andres Bello (Acta 002/2018). Upon obtaining a fresh cadaver, the liver, kidney, heart, lung, muscle, gonad, and adrenal gland were isolated, rinsed with ice-cold phosphate saline buffer (PBS), cut into small pieces, and protein extracts prepared using RIPA buffer (50 mM Tris pH 8.0, 150 mM NaCl, 0.1% SDS, 1% Triton X-100, 0.5% sodium deoxycholate) with protease inhibitors. Samples were homogenized with 20 dounce strokes and maintained for 30 min at 4 °C with rotation. Finally, samples were centrifuged for 20 min at 10,000× *g* at 4 °C. Supernatants were quantified, and 30 µg of protein were used for western blot assays using mouse anti-DeRep 120, mouse anti-DeRep 121, or mouse anti-α tubulin. Samples from *Cavia porcellus* liver and HEK293T cells were prepared as described above. For comparison between degu, HEK293T and guinea pig , 60 µg of protein were used for western blot assays.

To analyze the expression of FLAG-DeRep in HEK293T cells co-transfected with pcDNA3xFLAG-DeRep and pdBMVp or in NIH3T3 cells stably expressing FLAG-DeRep, cells were lysed using RIPA buffer, and 10 µg of each sample were used for western blot assays with anti-FLAG or anti-α tubulin antibodies.

To analyze the expression of viral proteins upon MVM infection, NIH3T3 cells expressing FLAG-DeRep or the control stable cell line were seeded at 5 × 10^4^ cells/well in 6-well plates, and 24 h later they were infected with a ¼ MVM dilution and lysed at 0, 12, 16, 20, or 24 h post infection, and western blots were performed using a rabbit anti-NS1/NS2 antibody (kindly donated by Dr. Peter Tattersall), mouse anti-FLAG, or rabbit anti-GAPDH antibody [6C5] (Abcam, Cambridge, UK).

### 2.8. RNA Extraction and PCR Amplification

Upon obtaining a fresh cadaver, the liver, kidney, heart, lung, muscle, gonad, and adrenal gland were isolated, rinsed with ice-cold phosphate saline buffer (PBS), cut into small pieces, and the RNA extracted with Trizol. One microgram of RNA was used for cDNA synthesis using the iScript gDNA Clear cDNA Synthesis Kit (Bio-Rad, Hercules, CA, USA) according to the manufacturer’s instructions. cDNA was PCR amplified and analyzed as described in [22].

### 2.9. Immunofluorescence Assays

To analyze the DNA damage marker γH2AX upon MVM infection, NIH3T3 cells expressing FLAG-DeRep or the control stable cell line were seeded at 2.5 × 10^4^ cells/well in 12 mm coverslips and infected with a ¼ MVM dilution for 24 h. Cells were rinsed twice in ice-cold PBS, fixed for 20 min in a freshly prepared solution of 4% paraformaldehyde in PBS, and washed 3 times with PBS; they were permeabilized for 5 min with 0.2% Triton X-100 in PBS, and after 3 rinses in PBS, were incubated in 1% BSA in PBS for 30 min at 37 °C, followed by an overnight incubation at 4 °C with rabbit anti-DYKDDDDK Tag (1:1000; Cell Signalling Cat#14793) and mouse anti-p-Histone H2A.X antibody (Ser 139) (1:1000; Santa Cruz Biotechnology). Cells were washed 3 times with PBS, then incubated with Alexa-conjugated secondary antibodies (ThermoFisher, USA) for 30 min at 37 °C. Cells were washed 3 times with PBS and incubated with NucBlue (ThermoFisher, Waltham, MA, USA) for 15 min. Coverslips were mounted with Fluoromont-G (Electron Microscopy Sciences, Hatfield, PA, USA) and analyzed by confocal laser microscopy (Nikon C2+, Melville, NY, USA). Triple-color immunofluorescent images were captured by multitracking imaging of each channel independently to eliminate possible crosstalk between fluorochromes. Images were analyzed using NIH ImageJ software. The quantification of fluorescence was carried out under threshold conditions, measuring signal intensity in a defined ROI.

### 2.10. MVM Infection Assays

To titrate MVM obtained in HEK293T cells expressing FLAG-DeRep or control cells, NIH3T3 (4 × 10^3^ cells/well) were seeded in 96-well plates. Twenty-four hours later, cells were infected with two-fold serial dilutions of each virus in quadruplicate for 1 h. The media was replaced, and cells were incubated in DMEM with 10% FBS for five days. Cells were fixed with 10% formaldehyde for 1 h at room temperature, washed, and stained with 1% crystal violet for at least 6 h. The stain was dissolved in 1% SDS, and its absorbance was measured at 595 nm.

To challenge cells with MVM, NIH3T3 cells stably expressing FLAG-DeRep or control cells were seeded in 24-well plates (3 × 10^4^ cells/well), and 24 h later, they were infected in triplicate with a two-fold serial dilution of MVM in complete media for 5 h. The media was then changed, and cells were incubated for five days in complete media. Cells were fixed and stained as above.

### 2.11. Chromatin Immunoprecipitation (ChIP)

NIH3T3 cells were seeded in 150-mm plates with a 3 × 10^6^ cells/mL density. Twenty-four hours later, cells were transfected with 30 µg of pcDNA3xFLAG-DeRep or empty vector, and 24 h after that, they were infected for 2 h with MVM diluted 1/8 in PBS. After changing the media, cells were cultured for 48 h and processed for ChIP according to [31] with modifications. Cells were crosslinked by incubation in DMEM with 1% FBS and 1% formaldehyde for 10 min at 37 °C. Crosslinking was stopped with 0.125 M of glycine. Cells were washed with ice-cold PBS, and Farnham lysis buffer (5 mM PIPES pH 8.0, 85 mM KCl, 0.5% NP-40) was added. Cells were collected, centrifuged at 1000× *g* for 5 min at 4 °C, and the cell pellet was resuspended in 1 mL of Farnham lysis buffer, shred 20 times using a 20-gauge needle, and centrifuged at 1000× *g* for 5 min at 4 °C. The pellet was resuspended in 500 µL of RIPA buffer (1x PBS, 1% NP-40, 0.5% sodium deoxycholate, 0.1% SDS). The suspension was sonicated in Qsonica Q800R3 for 15 min and then centrifuged at 13,000× *g* for 15 min at 4 °C. Four hundred microliters of the supernatant were mixed with 2 µg of anti-FLAG M2 or normal mouse IgG (Santa Cruz) for 1 h at 4 °C, and antibodies were sedimented with a mix of Dynabeads Protein A/G for 2 h at 4 °C. Beads were washed 5 times at 4 °C with 100 mM Tris pH 7.5, 500 mM LiCl, 1% NP-40, and 1% sodium deoxycholate. Crosslinking was reverted over night at 65 °C. The samples were treated with 1 μg/mL proteinase K for 1 h at 37 °C. DNA was purified using gel and PCR clean-up columns (Machery-Nagel) and was used for qPCR assay with the Brilliant II SYBER Green kit and primers directed to MVM NS (NS-F 5′-ACCAGCCAGCACAGGCAAATCTATTAT-3′; NS-R 5′-CATTCTGTCTCTGATTGGTTGAGT-3′) or VP (VP-F 5′-AAATTACTGCACTAGCAACTAGAC-3′, VP-R 5′-CTTCAGGAAAGGTTGACAGCA-3′).

### 2.12. Statistical Analysis

Values are presented as the mean ± standard deviation (SD) for 3 or more independent experiments. Statistical analyses with the Student’s *t* test were performed. Values of *p* < 0.05 were considered statistically significant. All statistical analyses were performed using Graphpad Prism (GraphPad Software Inc., San Diego, CA, USA).

## 3. Results

### 3.1. Generation of DeRep-Specific Antibodies

**Figure 1:**
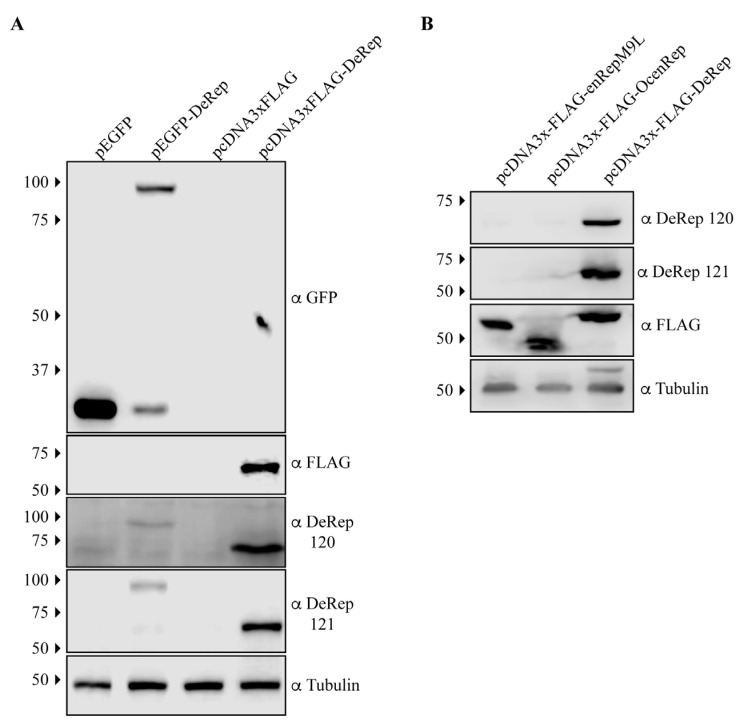
Validation of anti-DeRep antibodies by western blot assays. (**A**) NIH3T3 cells were transfected with plasmids encoding GFP-DeRep, FLAG-DeRep, or empty vectors. Forty-eight hours after transfection, cells were lysed, and western blots were performed using anti-GFP, anti-FLAG, anti-DeRep 120, or anti-DeRep 121. (**B**) NIH3T3 cells were transfected with plasmids encoding FLAG-enRepM9L, FLAG-OcenRep, or FLAG-DeRep. Forty-eight hours after transfection, cells were lysed, and western blots were performed using anti-FLAG, anti-DeRep 120, or anti-DeRep 121. Tubulin was used as a loading control. A representative experiment of at least three independent assays is shown. The migration of the molecular weight marker is indicated on the left-hand side. The antibodies used in each western blot are indicated on the right-hand side.

*EPV-Dependo.43-ODegus* is an EPV with an open reading frame of 1527 bp that encodes a protein of 508 amino acids, named DeRep. DeRep has a 62% amino acid identity to AAV2 Rep [22] and a high similarity to other AAV Rep proteins (Figure S1). We have previously shown that *EPV-Dependo.43-ODegus* (Odegus-4 in our previous report) is transcribed in at least 2 organs in degus: the liver and lung [22]. To determine if it is also expressed in vivo, we generated antibodies in mice using a bacterially produced DeRep protein. To test if the antibodies worked on western blot assays, we transfected cell lines with plasmids encoding full-length tagged GFP-DeRep or FLAG-DeRep (Figure 1A). When western blots were performed using anti-GFP antibodies, we detected the presence of GFP when the cells were transfected with the pEGFP vector or the fused protein GFP-DeRep when the cells were transfected with pEGFP-DeRep (Figure 1A, upper panel). Using the anti-FLAG antibody, we detected only one band in cells transfected with pcDNA3xFLAG-DeRep (Figure 1A, second panel). When custom-generated anti-DeRep 120 and anti-DeRep 121 were used, we detected both GFP-DeRep and FLAG-DeRep (Figure 1A, third and fourth panels), showing that both antibodies detect DeRep in western blot assays when it is expressed in cells.

We also tested if the anti-DeRep antibodies were able to recognize other NS-derived EPVs we have cloned in the same FLAG-expressing vector. We have previously described the transcription and expression in heterologous systems of enRepM9L, an intact EPV found in guinea pigs [21], and include in this analysis an intact EPV cloned from rabbits (*Oryctolagus cuniculus*) named OcenRep (unpublished data). When a western blot was performed, both anti-DeRep antibodies only recognized DeRep (Figure 1B, first and second panels), while the anti-FLAG antibody recognized the three FLAG-tagged proteins at their expected size. This shows that when expressed in cells, both antibodies specifically detect the expression of DeRep and no other EPV proteins.

### 3.2. DeRep Expression in Degu

**Figure 2:**
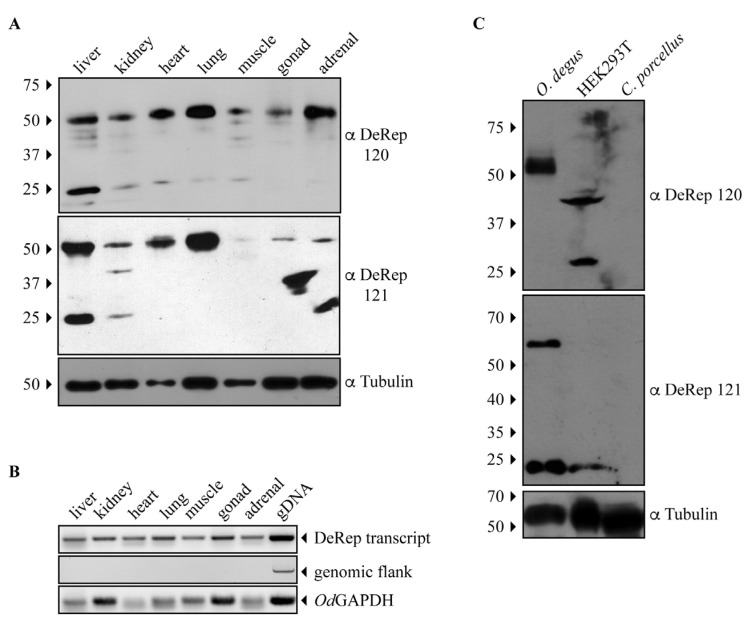
The protein DeRep is expressed in several organs of the degu. (**A**) Different organs of degu were isolated, lysed, and analyzed by western blot using the anti-DeRep 120 (upper panel) or anti-DeRep 121 (middle panel) antibodies. Samples from one representative animal of the six analyzed are shown. Tubulin was used as a loading control. The migration of the molecular weight marker is indicated on the left-hand side. The antibodies used in each western blot are indicated on the right-hand side. (**B**) Different organs of degu were isolated, lysed, and RNA extracted to synthesize cDNA. cDNA was PCR amplified with primers aligning inside the DeRep open reading frame (DeRep transcript), flanking *EPV-Dependo.43-ODegus* (genomic flank), or GAPDH transcript. Amplicons were detected in the agarose gel. One representative animal of the three analyzed is shown. (**C**) Lysates from *O. degus* (adrenal), HEK293T cells, and *C. porcellus* (liver) were analyzed by western blot using the anti-DeRep 120 (upper panel), anti-DeRep 121 (middle panel), and tubulin (lower panel) antibodies. Samples from one representative independent experiment of the three conducted are shown. The migration of the molecular weight marker is indicated on the left-hand side. The antibodies used in each western blot are indicated on the right-hand side.

Since both anti-DeRep antibodies can specifically detect DeRep when expressed in cells, we moved on to test if it was possible to detect DeRep in protein extracts obtained from degu tissues. Both antibodies showed similar results: a main band at the expected migration of DeRep (Figure 2A). Unexpectedly, this band was present in all tissue samples analyzed, not only from the liver and lungs. Thus, we revisited the previously analyzed animals and performed RNA extraction in some of the new animals, increasing the starting concentration of RNA for cDNA preparation and finding that the RNA is transcribed in all analyzed tissues (Figure 2B). We also found that both antibodies also detected a second band around 25 kDa, but mainly in liver samples (Figure 2A). To further test the specificity of our antibodies, we performed a second set of western blot assays, comparing one tissue of a different degu from that shown in Figure 2A with lysates from HEK293T cells and *Cavia porcellus* (guinea pig), a relatively closed rodent where we have shown that an intact Rep-derived EPV is transcribed in several tissues [21]. The adrenal sample from degu shows a similar pattern to that observed in Figure 2A with both antibodies; HEK293T shows a non-specific binding with antibody 120 and a weak signal around 25 kDa with antibody 121; more importantly, the liver samples from *C. porcellus* do not show any signal with either antibody (Figure 2C). These results indicate that the DeRep protein is expressed in different tissues of degu, which strongly suggests a functional role for it.

### 3.3. DeRep Blocks Exogenous Parvovirus Replication

It has not been reported if EPVs have functional roles in their hosts. As a first approach and considering that DeRep has NS/Rep characteristics (Figure S1), it is located in the nucleus when expressed in cell lines [21], and several EVE have a role in immunity [15,32,33], we asked if DeRep could affect parvoviral replication. The virus we decided to use was the ‘minute virus of mice’ (MVM), a cytolytic autonomous protoparvovirus with 37% homology of the NS1 protein to DeRep.

**Figure 3:**
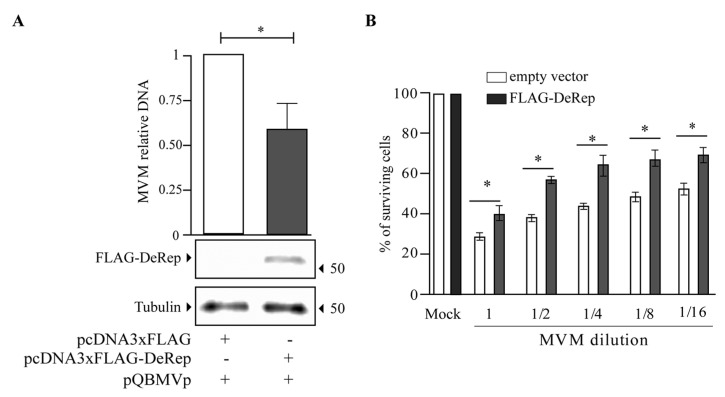
MVM production is reduced in the presence of FLAG-DeRep. (**A**) Quantification of viral DNA in HEK293T cells co-transfected with an empty vector or a FLAG-DeRep coding vector and the MVM molecular clone pQBMVp. The average of three independent experiments is shown. The expression of FLAG-DeRep was confirmed by western blot with an anti-FLAG antibody; tubulin was used as a loading control. The migration of the molecular weight marker is indicated on the right-hand side. The migration of FLAG-DeRep and tubulin is indicated on the left-hand side. (**B**) Infectivity of MVM produced in control (white bars) or FLAG-DeRep (gray bars)-expressing cells. NIH3T3 cells were infected with identical dilutions of each virus, and cell survival was quantified five days post-infection. The average of four independent experiments is shown. * *p* < 0.05.

**Figure 4:**
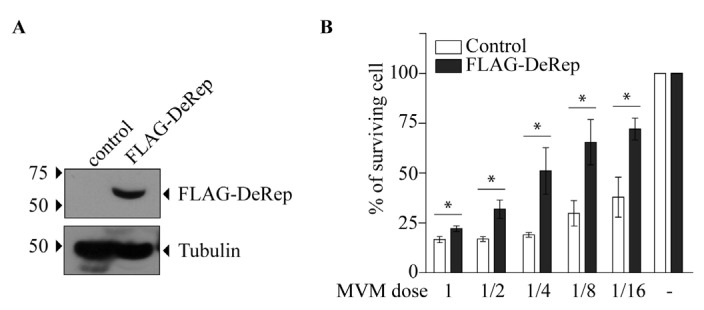
FLAG-DeRep reduces MVM replication in NIH3T3 cells. NIH3T3 cells expressing FLAG-DeRep in a stable manner or stably transfected with an empty vector (control) were generated and then infected with different dilutions of MVM. (**A**) Western blot showing the expression of FLAG-DeRep and its absence in the control cell line using the anti-FLAG antibody. The migration of molecular weight markers is indicated on the left-hand side. The migration of FLAG-DeRep and tubulin is indicated on the right-hand side. (**B**) Infectivity of MVM in control (white bars) or FLAG-DeRep (gray bars)-expressing cells. The average of five independent experiments is shown. The results are expressed as a percent of surviving cells, where 100% are non-infected cells. * *p* < 0.05.

Our first approach was to analyze the production of MVM in HEK293T cells co-transfected with the MVM molecular clone and the plasmid encoding FLAG-DeRep versus cells co-transfected with the empty vector. We found a significant reduction in viral DNA production in cells expressing FLAG-DeRep (Figure 3A) compared to control cells. When this experiment was performed to co-transfect the retroviral restriction factor TRIM5α [34], we found that viral DNA production has similar levels to that observed in cells co-transfected with the empty vector, while a reduction of around 50% is found in cells co-transfected with FLAG-DeRep (Figure S2). In line with this observation, we performed an infection assay and found that the virus prepared in cells that express FLAG-DeRep was significantly less infective than the virus prepared in cells that do not express it (Figure 3B). This suggested that DeRep could reduce MVM production from the molecular clone. We asked if DeRep could also reduce the replication of MVM upon infection. For this, we generated a stable NIH3T3 cell line that expresses FLAG-DeRep or its control version (Figure 4A) and challenged them with different dilutions of MVM. We found that DeRep can significantly protect cells from the cytopathic effect of MVM replication, even at high doses (Figure 4B). These results strongly suggest that DeRep can inhibit parvoviral replication.

**Figure 5:**
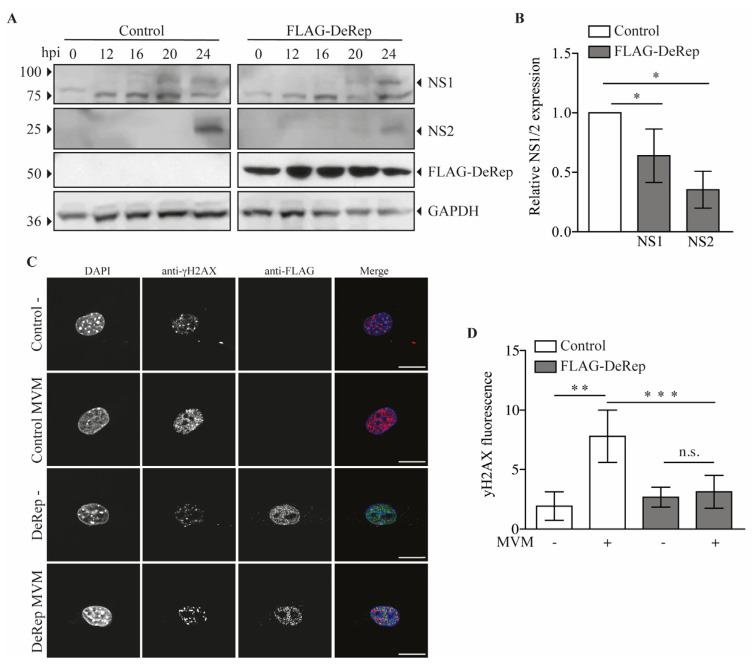
Expression of FLAG-DeRep reduces MVM protein expression and DNA damage induction in MVM-infected cells. (**A**) Control cells or cells stably expressing FLAGDeRep were infected with a ¼ MVM dilution and harvested at 0, 12, 16, 20, and 24 h post-infection. Cells were lysed, and western blot assays were performed using anti-NS1/2 serum (first and second panels), anti-FLAG (third panel), and anti-GAPDH (fourth panel). A representative western blot of four independent experiments is shown. (**B**) The NS1 and NS2 protein levels at 24 h post-infection were quantified and expressed relative to the loading control. (**C**) Control cells or cells stably expressing FLAG-DeRep seeded in coverslips were infected or not with a ¼ MVM dilution for 24 h. Cells were fixed and stained with mouse anti-γH2AX and rabbit anti-FLAG, followed by anti-mouse Alexa-546, anti-rabbit Alexa-488, and DAPI. A representative cell of 20 quantified cells is shown for each condition. (**D**) Quantification of γH2AX fluorescence labels in control and FLAG-Derep expressing cells the infection status in indicated in the X axis. Scale bar = 20 nm. * *p* < 0.05, ** *p* = 0.002, *** *p* < 0.001, n.s. = not significant.

Since we observed a reduction in viral DNA production in HEK293T cells when they were co-transfected with FLAG-DeRep, we wondered if viral protein production is also affected in FLAG-DeRep-expressing cells when they are infected with MVM. To test this, we performed a western blot assay with samples of cells infected with a single dose of MVM over a time course of 0, 12, 16, 20, and 24 h. We observed that in DeRep-expressing cells there is a delay in NS1 expression; in control cells, it is possible to detect NS1 at 16 h post-infection (hpi), and a clearly defined band is observed at 20 and 24 hpi, while in FLAG-DeRep, we observed a faint band at 20 hpi and a clear band at 24 hpi (Figure 5A). For NS2, we observed a band only at 24 hpi in both cell lines, but much less defined and intense in FLAG-DeRep (Figure 5A). We quantified the western blot signals for NS1 and NS2 at 24 hpi and found a significant reduction in both proteins in cells expressing FLAG-DeRep compared to control cells at the same time point (Figure 5B). In addition, we used immunofluorescence to analyze the presence of the DNA damage marker γH2Ax, which increases with the DNA damage that occurs in cells when MVM replicates [35]. We infected the cells and fixed them at 24 hpi. We then found that in control cells there is a significant increase in the signal of γH2AX upon infection compared to non-infected cells (Figure 5C,D), while in FLAG-DeRep-expressing cells a slight, but non-significant, increase in the γH2Ax signal is observed between non-infected and infected cells. When we compare control and FLAG-DeRep-infected cells, we clearly observe a reduced γH2AX signal in FLAG-DeRep-expressing cells (Figure 5C), which is significantly lower than that in the control cells (Figure 5D). Altogether, these results indicate that although MVM can replicate in cells expressing FLAG-DeRep, its replication is significantly reduced compared to control cells, suggesting a possible antiviral role for DeRep in its host.

**Figure 6:**
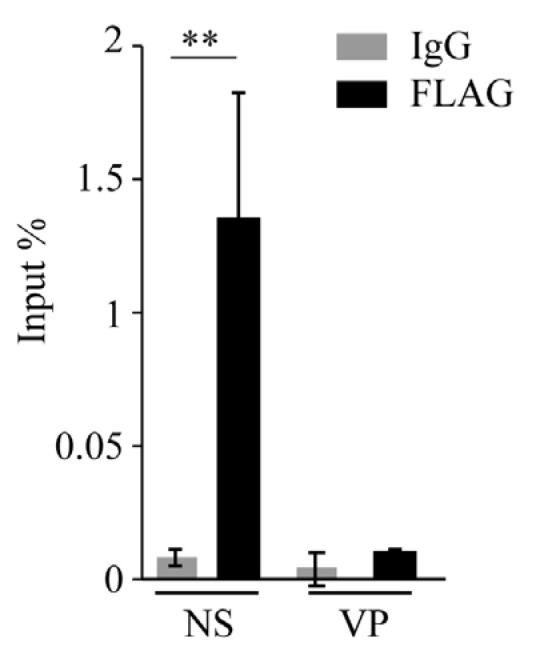
FLAG-DeRep binds to the MVM genome. NIH3T3 cells expressing FLAG-DeRep were infected for 48 h with MVM. Cells were fixed, and chromatin immunoprecipitation was performed with anti-FLAG or non-specific IgG. Immunoprecipitated DNA was recovered and analyzed by qPCR with primers against the NS or VP genes. Results are presented as a percentage of input recovered. ** *p* = 0.0094.

We observed that the Rep-derived protein DeRep is in the nucleus [21] (Figure 5C), the replication site of MVM. We therefore hypothesize that it could be bound to viral DNA to block its replication and/or transcription. To test whether DeRep is able to bind viral DNA, we performed chromatin immunoprecipitation assays in FLAG-DeRep cells infected with MVM. We found that FLAG-DeRep binds specifically to viral DNA in the NS region but not the VP region (Figure 6). These results are consistent with a role for DeRep as a dominant negative inhibitor of parvovirus replication. Further experiments are needed to determine whether it can form an inactive or partially active complex with the replication machinery or if it is blocking the binding of the replication and/or transcription machinery to viral DNA.

## 4. Discussion

Intact EPVs have been found at a surprisingly high frequency in mammalian genomes, suggesting that their conservation is associated with a potential physiological role in their host [1,6,21,22]. We and others have found EPVs derived from the Rep genes of dependoparvoviruses that contain intact ORFs and are transcribed in their hosts [21,22,27]. Here we show that one of these EPVs is translated within its host, the degu. Moreover, we have previously reported the expression in vitro of a fusion protein derived in part from an EPV in guinea pigs (*Cavia porcellus*) [21]. Here, however, we demonstrate the expression of a completely EPV-derived protein in vivo; to our knowledge, this is the first such demonstration.

Since our previous study on *EPV-Dependo.43-ODegus* showed transcription in the liver and lung, and the EPV of elephants also showed transcription in the liver [27], we expected a discrete protein expression if it was expressed as protein. To our surprise, we found DeRep protein in all analyzed tissues using either of the two affinity-purified antibodies we developed, although at different expression levels and always more robustly expressed in the liver. We revisited the previously analyzed animals and performed RNA extraction in some of the new animals, increasing the starting concentration of RNA for cDNA preparation and finding that the RNA is transcribed in all tissues (Figure 2B), concomitant with our protein expression patterns. When these antibodies were used in *Cavia porcellus* protein extract, no band was detected with either antibody (Figure 2C). We are therefore confident that the protein detected for both antibodies around 56 kDa is DeRep and not an unspecific binding. We also observed a second band around 25 kDa in some of the degu tissues analyzed with both antibodies. This could be a non-specific band, but it is also possible that Odegus4 mRNA allows the translation of a second protein from an internal AUG codon in Kozak context. Parvoviruses have mainly two genes that can encode several proteins by different mechanisms, such as the use of an internal promoter, splicing, and leaky scanning mechanisms [36], and some of these proteins are in frame with the principal protein that these genes encode. When analyzing the mRNA sequence of DeRep, we identified two putative start codons in a Kozak context that are in frame with the full-length ORF of DeRep. Both can be translated into proteins of 220 or 209 amino acids, respectively, corresponding to the 25 kDa bands we observed. More experiments are required to determine if the smaller protein is also translated from DeRep mRNA by a different transcript from an internal promoter or by a leaky scanning mechanism.

We cloned the coding sequence of the full-length DeRep protein, a 56 kDa protein that localizes in the nucleus [21] (Figure 5C), which is the replication site of parvoviruses. When co-transfecting the DeRep coding sequence along the molecular clone of MVM, we observed a reduction in viral DNA production as well as a lower viral titer when compared to control cells (Figure 3). Similarly, when DeRep-expressing cells are infected with MVM, viral replication is significantly reduced (Figure 4), which correlates with the delay observed in viral protein production (Figure 5A,B). Since DeRep is derived from a Rep parvoviral gene and contains both the Rep (catalytic domain with DNA binding and endonuclease activity) and Parvo_NS1 (DNA helicase and ATPase activity) domains, we tested if it was able to bind viral DNA. We found that DeRep binds to the NS region of the MVM genome (Figure 6), therefore co-localizing with the replication machinery. This has also been observed for itEBLN, which can co-localize with Borna disease virus (BDV) replication factories in the nucleus, reducing BDV replication [15]. Similar to itEBLN, DeRep exhibits antiviral activity against an exogenous virus of the same family. Further experiments are needed to determine its exact mechanism of action, for instance, if DeRep interacts with NS1 and the influence of its localization on its antiviral activity. DeRep’s predicted nuclear localization signal (NLS) (Figure S1) must be compared to a real NLS. For now, we can speculate that if an interaction with NS1 is occurring, even in the absence of an NLS in DeRep, we could find DeRep in the nucleus upon infection, still blocking MVM replication. MVM NS1 has been shown to complement nuclear localization-deficient versions of itself [37], and other NS proteins shuttle between cytoplasm and nucleus [38,39], so a putative interaction with DeRep in the cytoplasm could move DeRep to the nucleus. However, if the interaction is only with DNA, blocking NS1 binding to it, an NLS-null DeRep should lose its antiviral activity.

One important limitation of our study is that we have expressed DeRep in mouse cells, and it is in this context that DeRep is able to reduce the replication of a model protoparvovirus, MVM. Since there is no cell line derived from *Octodon degu*, we do not know if the level of expression we achieve in our stable cell line reflects the physiological levels of DeRep in degu cells. If a degu cell line can be established, it would be important to test if they are susceptible and/or permissive to MVM. In this context, DeRep gain and loss of function experiments will show the protein’s physiological relevance in the host. So far, no degu parvoviruses have been described, but given the ubiquity of parvovirus infection among mammals, it is likely they exist. We can speculate that if the function of intact EPVs in the host is to act as antivirals, there might exist parvoviruses that have co-evolved with degu. To know this, it will be necessary to understand the diversity of viruses in nature as well as the interaction between native wild animals and domestic animals that can act as vectors of viruses that do not normally circulate in wildlife, such as canine parvovirus.

Other intact, non-retroviral EVEs have been co-opted to perform cellular functions that are not necessarily related to counteracting exogenous viruses. One example is human EBLN, where hsEBLN1 can act as lncRNAs regulating gene expression [18], while hsEBLN2 has acquired a mitochondrial localization signal and is now important to regulate cell survival [17]. If parvoviruses that infect degu in nature are extinct, then we can speculate that the conservation of the *EPV-Dependo.43-ODegus* locus through this rodent evolution is due to domestication to perform a new cellular function as a protein. Its capacity to bind MVM DNA and nuclear localization suggest that it could be participating in DNA metabolism, but unlike hsEBLN1, which functions as RNA, it could be doing it as a protein.

In captivity, degus can live up to 13 years. They are gregarious animals that are models of social behavior [40,41] as well as neurobiology since they are used to study the retina [42,43] and they develop an Alzheimer’s-like disease while aging [44,45,46]. Therefore, they are interesting models that can be genetically manipulated using adeno-associated viruses (AVVs) as delivery vectors. Although we tested replication of a protoparvovirus rather than a dependovirus, our results should be considered when deciding which delivery tools can be used in degu. In favor of using AAVs to manipulate degu, a preliminary assay showed that transduction by a GFP-coding AAV2 vector was not impaired in cells expressing DeRep. More experiments will be needed to demonstrate if there is a saturation phenomenon, as it happened for some retroviral restriction factors such as TRIM5alpha and Fv1 [47], or if AAV infection is indeed not affected by DeRep. It could also be possible that the different levels of DeRep observed in the different tissues analyzed can confer differential protection against parvoviruses or AAV vectors.

## 5. Conclusions

The endogenous parvovirus (EPV) locus ‘*EPV-Dependo.43-ODegus’* (Odegus4) is a host gene encoding an intact, Rep-derived protein that is expressed in vivo and named DeRep. This protein, when expressed in cell culture, is able to block parvovirus replication, which suggests an antiviral role.

## Data Availability

All data supporting this work is available upon request.

## Supplementary Materials

The following supporting information can be downloaded at: https://www.mdpi.com/article/10.3390/v15071420/s1. Figure S1. Multiple sequence alignment showing homology between DeRep protein and the NS proteins of representative dependoparvoviruses. Figure S2. MVM DNA is reduced in the presence of FLAG-DeRep but not in the presence of FLAG-TRIM5α.
