# Supplementary material for: Antiviral Activity of an Endogenous Parvoviral Element
Source: Viruses. 2023 Jun 23;15(7):1420. doi: 10.3390/v15071420 (PMC10384997; doi:10.3390/v15071420)
Supplement: Supplementary file 1 [file viruses-15-01420-s001.zip › viruses-2466904-supplementary.pdf]

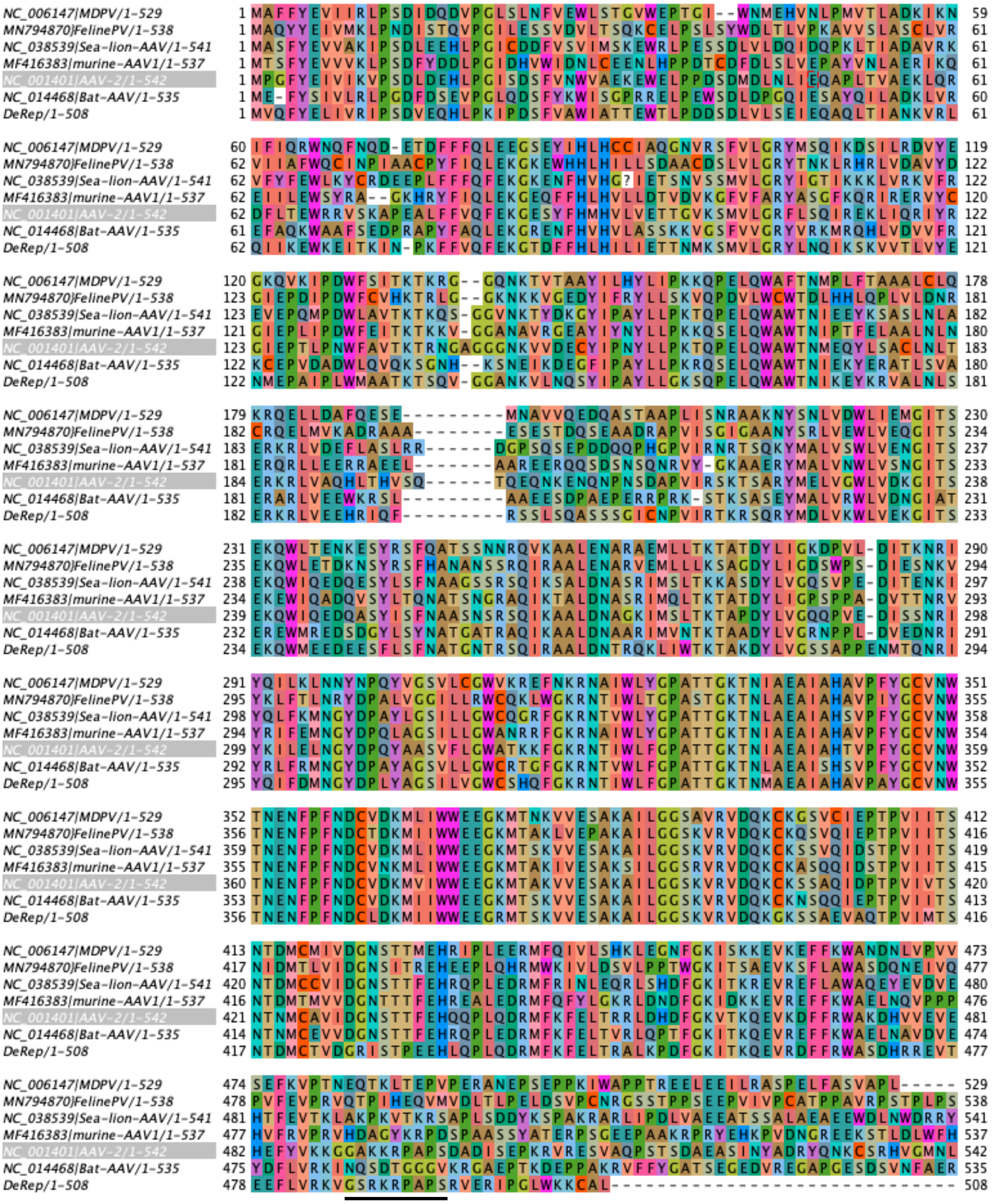

Figure Supplementary1. Multiple sequence alignment showing homology between DeRep protein and the NS proteins of representative dependoparvoviruses. Virus taxon labels include GenBank accession numbers virus, species names, and coordinate information. DeRep putative nuclear localization signal is underlined. AAV=adeno-associated virus.

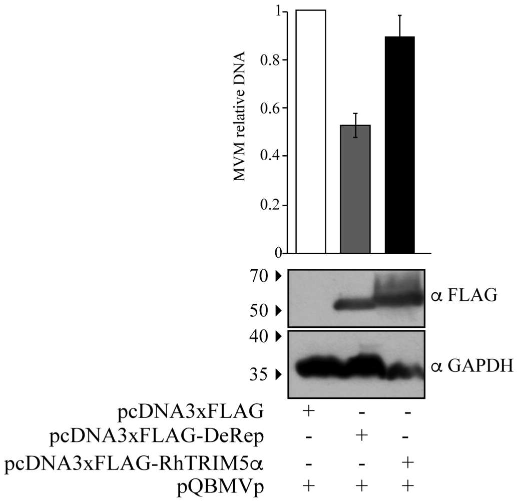

Figure Supplementary 2. MVM DNA is reduced in the presence of FLAG-DeRep but not in the presence of FLAG-TRIM5α. Quantification of viral DNA in HEK293T cells co-transfected with an empty vector, FLAG-DeRep or FLAG-TRIM5α (rhesus monkey) coding vector and the MVM molecular clone pQBMVp. The average of 2 experiments is shown. The expression of FLAG-DeRep and FLAG-TRIM5α was confirmed by western blot with anti-FLAG antibody, tubulin was used as loading control. The migration of molecular weight marker is indicated on the right-hand side.
